# *In Vitro* Inhibitory Effects of Cyandin-3-rutinoside on Pancreatic α-Amylase and Its Combined Effect with Acarbose

**DOI:** 10.3390/molecules16032075

**Published:** 2011-03-02

**Authors:** Sarinya Akkarachiyasit, Sirintorn Yibchok-Anun, Sumrit Wacharasindhu, Sirichai Adisakwattana

**Affiliations:** 1 Department of Pharmacology, Faculty of Veterinary Science, Chulalongkorn University, Bangkok 10330, Thailand; E-Mails: Akkara_sarin@hotmail.com (S.A.); Sirintorn.y@chula.ac.th (S.Y-A.); 2 Natural Products Research Unit, Department of Chemistry, Faculty of Science, Chulalongkorn University, Bangkok 10330, Thailand; E-Mail: Sumrit.w@chula.ac.th; 3 Department of Transfusion Medicine, Faculty of Allied Health Sciences, The Medical Food Research and Development Center, Chulalongkorn University, Bangkok 10330, Thailand; 4 Program in Nutrition and Dietetics, Faculty of Allied Health Sciences, Chulalongkorn University, Bangkok 10330, Thailand

**Keywords:** cyanidin-3-rutinoside, pancreatic α-amylase, acarbose, additive inhibition

## Abstract

The inhibitory activity on pancreatic α-amylase by cyanidin-3-rutinoside was examined *in vitro*. The IC_50_ value of cyanidin-3-rutinoside against pancreatic α-amylase was 24.4 ± 0.1 μM. The kinetic analysis revealed that pancreatic α-amylase was inhibited by cyanidin-3-rutinoside in a non-competitive manner. The additive inhibition of a combination of cyanidin-3-rutinoside with acarbose against pancreatic α-amylase was also found. These results provide the first evidence for the effect of cyanidin-3-rutinoside in a retarded absorption of carbohydrates by inhibition of pancreatic α-amylase which may be useful as a potential inhibitor for prevention and treatment of diabetes mellitus.

## 1. Introduction 

Pancreatic α-amylase is a key enzyme in the digestive system that catalyses the initial step in hydrolysis of starch to maltose and finally to absorbable glucose. Degradation of dietary starch leads to elevated postprandial hyperglycemia. Retardation of starch hydrolysis by inhibition of pancreatic α-amylase is one of therapeutic approaches for the control of postprandial hyperglycemia in pre-diabetes, diabetes and obesity [1]. Suppression of postprandial hyperglycemia subsequently delays the progression of micro- and macro-vascular complications such as microangiopathy, cardiovascular, and cerebrovascular diseases [2]. 

**Figure 1:**
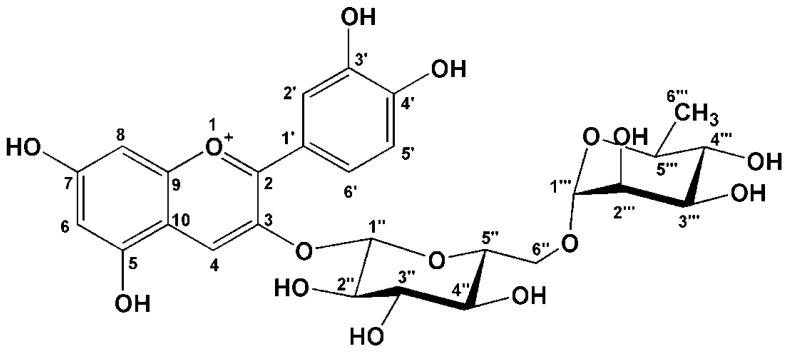
The structure of cyanidin-3-rutinoside.

Screening of inhibitors of carbohydrate digestive enzymes from natural products has been used for alternative prevention and treatment of type 2 diabetes mellitus. Anthocyanins are a class of flavonoids which are widely distributed in various human diets through crops, vegetables, and fruits. [3]. The health-promoting effects of anthocyanins are being increasingly exploited as nutraceuticals and dietary supplements. The biological activities of anthocyanins have been focused on their potential benefits such as antioxidative [4], anticancer [5], anti-inflammatory [6] as well as anti-diabetic activities [7]. Cyanidin-3-rutinoside (Figure 1) is a natural colorant found in litchi, black currant, capulin and sweet cherry [8,9,10]. It has shown that cyanidin-3-rutinoside selectively kills leukemic cells by induction of oxidative stress [11]. In addition, previous studies have shown α-glucosidase inhibitory activity of cyanidin-3-rutinoside against yeast and mammalian α-glucosidase [12,13]. Although the α-glucosidase inhibitory activity of cyanidin-3-rutinoside has been investigated, studies regarding the inhibitory effect of cyanidin-3-rutinoside against pancreatic α-amylase activity have not been undertaken to the best of our knowledge. 

The aim of current study was to investigate the inhibitory effect of cyanidin-3-rutinoside against pancreatic α-amylase and to evaluate the types of kinetic inhibition on pancreatic α-amylase. Furthermore, the combined effect of acarbose and cyanidin-3-rutinoside was also investigated *in vitro*. 

## 2. Results and Discussion 

### 2.1. The IC_50_ values for pancreatic α-amylase 

C3R strongly suppressed pancreatic α-amylase activity in a concentration-dependent manner (Table 1). As shown in Table 2, the IC_50_ value of C3R against pancreatic α-amylase was 24.4 ± 0.1 μM, which was lower potency than acarbose (18.1 ± 0.1 μM).

In recent years, the search for new chemical compounds as potential pancreatic α-amylase inhibitors with a high specific affinity has intensified. Pancreatic amylase inhibitors are also known as starch blockers because they contain substances that prevent dietary starch from being absorbed by the body. It digests and catalyses the initial step in hydrolysis of starch to maltotriose, maltose, and limit dextrins. Digestion of the limit dextrins and disaccharides, both dietary and starch-derived, to monosaccharides such as glucose, galactose, and fructose is accomplished by intestinal α-glucoidases located on the luminal surfaces of enterocytes lining the microvilli of the small intestine. This is the first study to investigate the inhibitory effect of C3R on porcine pancreatic α-amylase. Moreover, we earlier reported the inhibitory effect of C3R against intestinal α-glucosidase both maltase and sucrase *in vitro* [13]. The results showed that there was quite selective inhibition on intestinal sucrase because the IC_50_ value for intestinal maltase activity was much higher than that of sucrase activity. Nevertheless, C3R was much less potent than that of acarbose on the intestinal maltase and sucrase inhibition. Interestingly, the structure-activity relationship of cyanidin and its glycosides against pancreatic α-amylase and intestinal α-glucosidase was also reported in our previous study [13,14]. It found that the presence of glucose moiety at the 3-*O*-position of cyanidin markedly increases the potency of pancreatic α-amylase inhibition whereas the replacement of 3-*O*-glucose of cyanidin-3-glucoside by galactose residue directly affects to decrease pancreatic α-amylase activity [14]. In contrast, one of interesting findings is that the replacement of 3-*O*-glucose of cyanidin-3-glucoside by galactose residue directly affects to increase intestinal sucrase inhibitory activity. In fact, the structure of glucose and galactose, molecules have the same molecular formula but different structural formulae concerning the position of the hydroxyl (-OH) group on C-4. It can be assumed that the structural difference of the sugar at the 3-*O*-position may play an important factor for modulating the inhibition of intestinal sucrase and pancreatic α-amylase [14]. When comparing the IC_50_ values of C3R and cyanidin glycosides from a previous study, C3R is the most potent pancreatic α-amylase and intestinal sucrase inhibitor among cyanidin and its glycosides. It suggests that the introduction of a disaccharide (rutinose) in the 3-*O*-position of cyanidin may play a more important role for increasing pancreatic α-amylase and intestinal sucrase inhibitory activity than the presence of monosaccharide. However, the presence of sugar at 3-*O*-position of cyanidin results in the reduction of free-radical scavenging ability. It has been found that an increase in the polarity of the compound by adding monosaccharide or dissacharide affects the access of free radical scavenging potency in DPPH assay. The order of potency was cyanidin > cyanidin-3-glucoside > cyanidin-3-rutinoside ≅ cyanidin-3-galactoside, which differs from the potency order for pancreatic α-amylase inhibition [15]. It suggests that different sugar molecules may provide different molecular structures in which they may either enhance or diminish the potency of biological activities.

It has been established that an increase in postprandial hyperglycemia could contribute to the increase of hemoglobin glycosylation (HbA1c) by up to 25% in inadequately controlled patients with type 2 diabetes [16]. The decrease in HbA1c level could reduce the incidence of chronic vascular complications in diabetic patients [2]. Considerable amounts of anthocyanins are ingested as constituents of the human diet, 180–215 mg daily [17]. C3R is a natural colorant found in red sweet cherry, blackcurrant and other fruits. For example, red sweet cherry (Kordia) contains C3R, as a major anthocyanin, yielding at 184 mg/100 g fresh weight [18]. Moreover, it has been found that the abundant anthocyanins in mulberry pigment are cyanidin 3-*O*-rutinoside (60%) and cyanidin 3-*O*-glucoside (38%) [19]. It can be assumed that consumption of C3R-enriched fruits may suppress postprandial hyperglycemia through inhibition of pancreatic α-amylase and α-glucosidase, consequently, helping to the prevention of diabetic complications by decreasing HbA1c level. However, clinical data on consumption of C3R-enriched fruits in diabetic patients are limited. The further experiment of C3R-enriched fruits is required to evaluate its clinical efficacy for potential application in pre-diabetic or diabetic rats.

### 2.2. The type of inhibition of cyanidin-3-rutinoside on pancreatic α-amylase

**Figure 2:**
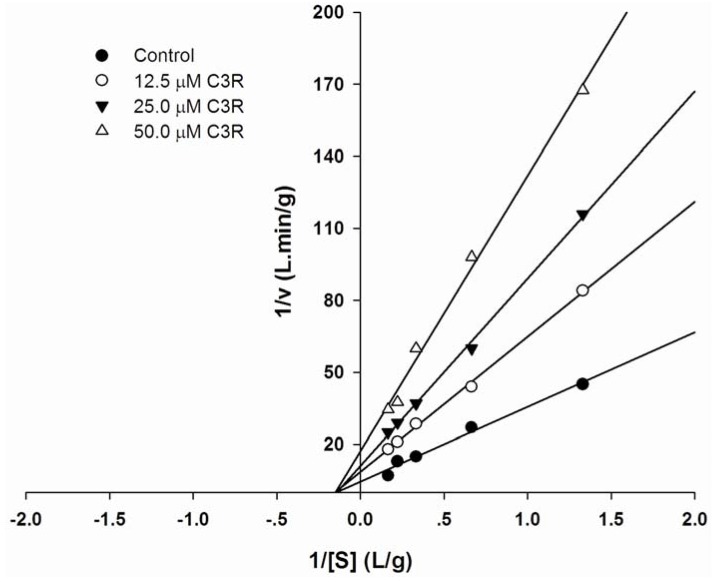
Lineweaver-Burk plots for inhibitory activity of C3R on pancreatic α-amylase.

To further investigate the inhibitory characteristics of C3R, a kinetic inhibition study was performed using Lineweaver-Burk double reciprocal plots. As shown in Figure 2, Lineweaver-Burk plot of C3R generated straight lines which had same intersections on X-axis, indicating that C3R activity was of a non-competitive type. The binding mode of C3R was assumed to be that one inhibitor can bind either to active site of free enzyme or to the enzyme-substrate complex with an equal affinity for binding. In addition, C3R can bind at the site on pancreatic α-amylase. It has recently been shown the pancreatic α-amylase inhibitory activity of flavonols and flavones is associated with hydrogen bonds between the hydroxyl groups of the polyphenol ligands and the catalytic residues of the binding site, leading to formation of a conjugated pi-system that stabilizes the interaction with the active site [20]. Although the molecular interaction of C3R on specific binding site on pancreatic α-amylase remains unknown but the information of flavonols and flavones mentioned above, it can be hypothesized that cyanidin and its glycosides may interact with protein by using hydroxyl groups in its molecular structure to form hydrogen bonds with the polar groups (amide, guanidine, peptide, amino and carboxyl groups) of amino acid residues in the active site of the pancreatic α-amylase by covalent and/or non-covalent interaction. To prove this hypothesis, using a computer modeling of docking structure to identify the binding activity of C3R on pancreatic α-amylase is needed to further investigation. 

### 2.3. The combined effect of cyanidin-3-rutinoside with acarbose on inhibition of pancreatic α-amylase activity in vitro

**Figure 3:**
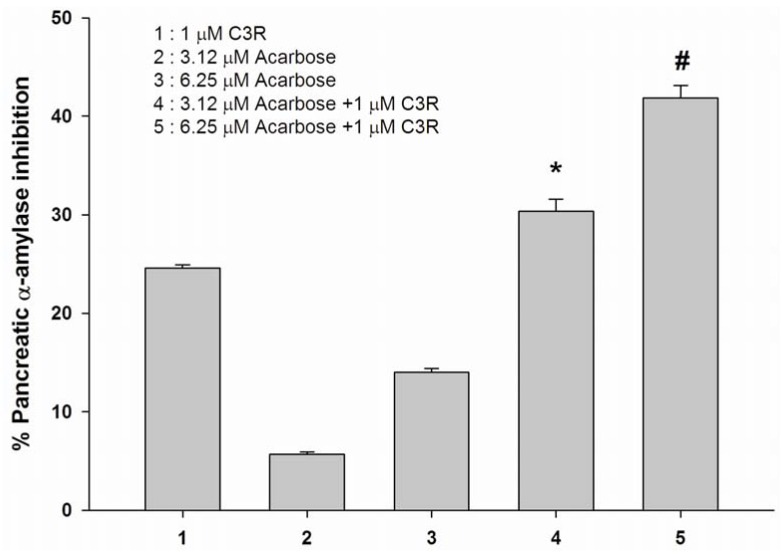
The combined effect of acabose and C3R (1 μM) on inhibition of pancreatic α-amylase. Result are expressed as means ± S.E.M; *n* = 3. *P < 0.01 compared with acarbose (3.12 μM) and ^#^*P* < 0.01 compared with acarbose (6.25 μM).

The assay was then performed in the solution containing acarbose alone or in mixtures with C3R (1 μM) When C3R was added to the assay system containing acarbose (3.12 μM and 6.25 μM), the percentage pancreatic α-amylase was increased as compared to acarbose alone (Figure 3). The results showed that the percentage inhibition of mixtures was equal to the sum of acarbose and C3R, suggesting that the combination of C3R and acarbose produced an additive inhibition. *Acarbose* is an anti-diabetic drug used to treat type 2 diabetes mellitus and, in some countries, pre-diabetes. A recent report has shown that treatment of acarbose (300 mg/day) was associated with a 25% reduction in the incidence of diabetes in subjects with impaired glucose tolerance [21]. Administration of acarbose (300 mg/day) is associated with a 20% reduction of the peak of postprandial hyperglycemia which helps to prevent glucotoxicity and the consequent hyperinsulinaemia [22]. 

Clinical data reveals that the HbA1c level was reduced by 0.77% in diabetic patients after intake of 50 mg acarbose three times daily [23]. Combination between acarbose and natural products may be one of alternative treatment of diabetes mellitus. Our present study reveals that combination of acarbose with C3R produces a significant additive inhibitory effect against pancreatic α-amylase. Moreover, our reports found that C3R shows a synergistic inhibition against intestinal maltase and sucrase when combined with acarbose [13]. It suggests that these effects would have significant clinical benefit of combination therapy for controlling postprandial hyperglycemia and reduction of HbA1c levels in diabetic patients. Furthermore, combined therapy with C3R may diminish the dose of acarbose, the progressive increase in optimal drug dosage, and costs associated with pharmaceutical disease management. 

## 3. Experimental 

### 3.1. Chemicals

Porcine pancreatic α-amylase and 3,5-dinitrosalicylic acid were purchased from Sigma Chemical Co. Ltd. (St. Louis, MO, USA). Acarbose was obtained from Bayer (Leverkusen, Germany). Cyanidin-3-rutinoside chloride (C3R) was synthesized from quercetin-3-rutinoside according to the previous method [24]. After purification, the chemical structure of C3R (Figure 1) was confirmed by using ^1^H-NMR, ^13^C-NMR and mass spectrometry data. All others chemicals used were of analytical grade.

### 3.2. Pancreatic α-amylase inhibition assay

The pancreatic α-amylase inhibition assay was performed according to the literature procedure with slight modificationsd [25]. Porcine pancreatic α-amylase was dissolved in 0.1 M phosphate buffer saline, pH 6.9. The various concentrations of cyanidin-3-rutinoside were added to solution containing in 1 g/L starch and phosphate buffer. The reaction was initiated by adding amylase (3 U/mL) to the incubation medium to a final volume of 500 μL. After 10 min the reaction was stopped by adding 0.5 mL dinitrosalicylic (DNS) reagent (1% 3,5-dinitrosalicylic acid, 0.2% phenol, 0.05% Na_2_SO_3_, and 1% NaOH in aqueous solution) to the reaction mixture. The mixtures were heated at 100 °C for 10 min and 500 μL of 40% potassium sodium tartarate solution was added to the mixtures to stabilise the colour. After cooling to room temperature in a cold water bath, absorbance (Abs) was recorded at 540 nm using spectrophotometer. Acarbose was used as a positive control for this assay. Cyanidin-3-rutinoside and acarbose were dissolved in DMSO. The percentage inhibition was calculated by follow the equation.

$$\%\;\mathrm{Inhibition}=\frac{\left[\Delta {\mathrm{Abs}}_{540\left(\mathrm{Control}\right)}-\Delta {\mathrm{Abs}}_{540\left(\mathrm{Sample}\right)}\right]}{\Delta {\mathrm{Abs}}_{540\left(\mathrm{Control}\right)}}\times 100$$

### 3.3. Enzyme kinetics 

In order to investigate the type of inhibition, the enzyme kinetic analysis was performed according to the above reaction. Maintaining the quantity of porcine pancreatic α-amylase constant at 3 units/mL and C3R (from 0.1 to 1.0 mM) was measured in various concentrations of starch. The type of inhibition was calculated on the basis of Lineweaver–Burk by reciprocally plotted data (substrate concentration on horizontal axis and velocity on vertical axis). 

### 3.4. Combined inhibitory effect of cyanidin-3-rutinoside and acarbose 

The various concentrations of acarbose were combined with or without C3R at low concentration. The reaction was performed according to the above assay. Results were expressed as the percentage inhibition of the corresponding control values.

### 3.5. Statistical analysis 

Data were expressed as means ± S.E.M. The IC_50_ values were calculated from plots of log concentration of inhibitor concentration versus percentage inhibition curves by using Sigma Plot 10.0 (IL, USA). Statistical analysis was performed by Student *t*’test. *P* < 0.01 was considered to be statistically significant.

## 4. Conclusions 

The present study shows that C3R markedly inhibits pancreatic α-amylase. The combined effect of acarbose and C3R is reported here for the first time. These results suggest that C3R may be potentially useful to control postprandial hyperglycemia in patients with type 2 diabetes through inhibition of intestinal α-glucosidase and pancreatic α-amylase. 

## Figures and Tables

**Table 1:** The percentage enzyme inhibition of cyanidin-3-rutinoside (C3R) on pancreatic α-amylase. Results are expressed as mean ± S.E.M., n = 3.

	**Concentration of cyanidin-3-rutinoside (αM)**						
		0.1	1.0	10.0	100.0	250.0	500.0	1000.0
% Inhibition	0.8 ± 0.1	23.7 ± 0.2	44.3 ± 0.1	52.1 ± 0.2	64.1 ± 0.3	68.1 ± 0.2	70.3 ± 0.3

**Table 2:** The IC_50_ values for pancreatic α-amylase, intestinal α-glucosidase (maltase and sucrase) by cyanidin-3-rutinoside and acarbose. Results are expressed as means ± S.E.M., n = 3. ^a^The IC_50_ value of cyanidin-3- rutinoside was previously reported in Adisakwattana *et al.* [13].

Compounds	IC_50_ values (μM)		
	Pancreatic α-amylase	Maltase	Sucrase
Cyanidin-3-rutinoside	24.4 ± 0.1	2,323 ± 14.8 ^a^	250.2 ± 8.1 ^a^
Acarbose	18.1 ± 0.1	2.7 ± 0.1 ^a^	29.6 ± 3.5 ^a^
